# Supplementary material for: The first leech body fossil predates estimated hirudinidan origins by 200 million years
Source: PeerJ. 2025 Oct 1;13:e19962. doi: 10.7717/peerj.19962 (PMC12495953; doi:10.7717/peerj.19962)
Supplement: Supplemental Information 1 [file peerj-13-19962-s001.pdf]

Supplementary Information

for

The first leech body fossil predates estimated hirudinidan origins by 200 million years

Danielle de Carle, Rafael Eiji Iwama, Andrew J. Wendruff, Loren E. Babcock, Karma Nanglu

Corresponding author: danielle.decarle@utoronto.ca

This file includes:

Supplementary Text: Notes on Character Coding

Supplementary Figures 1-6

References

Description of additional supplemental files

Other supplementary materials for this manuscript include the following:

Supplemental Files 1-7

Supplementary Text

<u>Notes on Character Coding</u>

Below are notes on characters added to the phylogenetic matrix. Further information is available in the matrix itself (**Supplemental File 1**).

56. Clitellum (present/absent): This character is a synapomorphy for Clitellata and was added to assist in resolving relationships between clitellate annelids

57. Clitellum externally visible (dependent on char. 56; present/absent): As above, this character assists in resolving relationships between clitellate annelids; although members of Hirudinea possess a clitellum, it is not generally not visible externally (Purschke et al., 1993).

58. Chaetae restricted to anterior-most segments (dependent on char. 30; present/absent): This is a synapomorphy for Acanthobdellida, the only hirudinean taxon that possesses chaetae (Sawyer, 1986).

59. Disc-like posterior attachment structure (present/absent): Among taxa included in our matrix, posteriorly-located disc-like structures used for attachment to hosts/substrate are common to Hirudinea and monogenean Platyhelminthes. This character was included to accommodate the hypothesis that *Macromyzon* might belong to Platyhelminthes.

60. Mechanism of attachment with posterior structure (hooks/suckers/chemical adhesive organs): The mechanism of action of these posterior attachment structures varies among the taxa sampled here: the haptors of monogenean flatworms use suction (suckers) and/or hooks; whereas the attachment structures of Acanthobdellida and Hirudinida also use suction, those of Branchiobdellida attach using adhesive secretions (Weigl, 1994). This character was included to differentiate between the morphologically similar, yet non-homologous, disc-like attachment structures of extant Hirudinea and Platyhelminthes.

61. Segments externally subdivided into annuli (dependent on char. 21; present/absent): Among the segmented taxa included here, only members of Hirudinea have segments which are subdivided into uniform ring-like annuli which are readily visible externally. These subdivisions are superficial only, and do not correspond to any internal structure (Sawyer, 1986).

62. Annulation pattern in midbody segments (biannulate/triannulate): Within an individual worm, the number of annuli per segment generally decreases toward the anterior- and posterior-most ends, but remains consistent in all mid-body segments (called somites). Branchiobdellidan somites are divided into two annuli, whereas acanthobdellidan and hirudinidan somites have three annuli each. In the latter two orders, these annuli may themselves be further subdivided over the course of development (Sawyer, 1986).

63. Pygidium (present/absent): This character describes the terminal segment found in polychaetes. This segment contains the anus, and is derived from the area posterior to the telotroch in developing polychaete larvae. It excludes the non-homologous pygidium of trilobites. The character aids in resolving annelid interrelationships: it is coded as present for all

polychaete taxa, and absent for all hirudinean taxa, in which the pygidium is lost and the posteriormost segments are fused to form the caudal sucker (Purschke et al., 1993). It is coded as absent for *Macromyzon siluricus*. as the terminal unit is the caudal sucker.

64. Anterior host-attachment structure (present/absent): Among taxa included in our matrix, anteriorly-located structures used for attachment to hosts/substrate are common to Hirudinea and cestode Platyhelminthes.

65. Mechanism of attachment with anterior structure (hooks/pinching (bothridia)/chemical adhesion/chaetae/suction): This character was included to differentiate between the morphologically similar, yet non-homologous, disc-like attachment structures of extant Hirudinea and Platyhelminthes. The mechanism of action of these anterior attachment structures varies among the taxa sampled here: cestode scolices use hooks and/or bothridia, which pinch host tissue; as with their posterior attachment structures, branchiobdellidans use adhesive secretions (Weigl, 1994) and leeches use suction (Sawyer, 1986); acanthobdellids, however, use anterior chaetae for attachment. The two acanthobdellidan species also have anterior suckers which, though less developed than those of leeches, likely function in the same manner as those of leeches (Bielecki et al., 2014). *Acanthobdella* uses its chaetae exclusively, while *Paracanthobella* also has an anterior sucker which appears leech-like, though the functionality of this sucker has not been assessed.

66. Pharyngeal jaws (present/absent): This character describes the jaw structures found in polychaetes and was included to aid in resolving annelid relationships. It does not include the introvert of some cycloneuralians (character 24), which is also an eversible feeding structure. It is coded as present for the polychaetes *Dryptoscolex*, *Harmothoe*, *Esconites* and *Eunice*. It is coded as absent in the polychaete *Capitella*. It is coded as present for *Branchiobdella* and *Hirudo* and absent for all other members of Hirudinea. It is coded as ? for Macromyzon, as the internal anatomy of the head region is not clearly preserved.

67. Jaw condition (dependent on char. 66; dorsoventral, hook-shaped/with mandibles and maxillae/dorsoventral, plate-shaped/tripartite): This character describes the morphology and orientation of the pharyngeal jaws. It was included to help resolve polychaete interrelationships, and reflect the differences between those structures as found in polychaetes, Branchiobdellida, and tooth-bearing Hirudinida, all three of which are likely non-homologous with one another. It is coded as "dorsoventral, hook-shaped" for *Harmothoe* and *Dryptoscolex*; it is coded as "with mandibles and maxillae" for *Eunice* and *Esconites*; it is coded as "dorsoventral, plate-shaped" for *Branchiobdella*; it is coded as "tripartite" for *Hirudo*; and it is coded as unknown for *Macromyzon*, as the internal anatomy of the head region is not clearly preserved (see character 66).

68. Chitinous microvillar appendages serially repeated (dependent on char. 30; present/absent): This character describes the serial repetition of chaetae found in Annelida, specifically the polychaetes and oligochaetes. Oligochaetes have subsequently lost the parapodia which are the chaetal attachment points in polychaetes, but retain the chaetae themselves. Uniquely among Hirudinea, Acanthobdellida retains chaetae, but they are restricted to two pairs

on each of five consecutive segments (*i.e*. III–VII) in the anterior region of the body (*i.e*. segments III–VII) (de Carle et al., 2022).

69. Midbody segments internally divided by complete septa (dependent on char. 21; present/absent): This character was included to help resolve relationships within Annelida, and particularly Clitellata. Leeches are characterized by complete reduction of septa throughout the body; Acanthobdellids have incomplete septa in some anterior and midbody segments, but not in the midbody; branchiobdellidan midbody segments are internally delineated by complete septa (Holt, 1965; Sawyer, 1986). It is coded as absent for *Acanthobdella*, *Hirudo*, and *Glossiphonia*, and present for all other annelids. It is coded as ? for *Macromyzon*, as this feature is not visible externally.

70. Male and female reproductive organs restricted to segments XI-XIII (present/absent): This is a clitellate-specific character, and was included to help resolve annelid relationships; although hirudineans have serially-repeated testisacs in some midbody segments, these derive from single, elongate testes which arise in segment XI and are subsequently elaborated (Sawyer, 1986). It is coded as present for all clitellates (*i.e.* Lumbriculidae, Branchiobdellida, Acanthobdellida, *Hirudo*, and *Glossiphonia*), absent for the extant polychaetes (*i.e. Harmothoe*, *Eunice*, and *Capitella*), and ? for the fossil taxa (*i.e. Macromyzon*, *Esconites*, and *Dryptoscolex*).

71. Condition of male pores in segments XI-XIII (dependent on char. 70; paired/fused): This clitellate-specific character was included to help resolve relationships within Clitellata. It is coded as paired for Lumbriculidae, and fused for members of Hirudinea (*i.e.* Branchiobdellida, Acanthobdellida, *Hirudo*, and *Glossiphonia*), which have a single external male genital opening (Purschke et al., 1993). It is coded as ? for *Macromyzon*.

72. Condition of female pores in segments XI-XIII (dependent on char. 70; paired/fused): As above, this character was included to help resolve relationships within Clitellata. It is coded as paired for Lumbriculidae and *Branchiobdella*, and fused for *Acanthobdella* and Hirudinida (*i.e. Hirudo* and *Glossiphonia*), which have a single external female genital opening (Purschke et al., 1993). It is coded as ? for *Macromyzon*.

73. Male opening in segment XI (dependent on char. 70; present/absent): This character was also included to help resolve relationships within Clitellata. It is coded as absent for all members of Clitellata except *Hirudo* and *Glossiphonia*, as the position of the external male genital opening is specific to true leeches (Purschke et al., 1993) It is coded as ? for *Macromyzon*.

74. Female opening in segment XII (dependent on char. 70; present/absent): This character was also included to help resolve relationships within Clitellata. It is coded as absent for all members of Clitellata except *Hirudo* and *Glossiphonia*, as the position of the external female genital opening is specific to true leeches (Purschke et al., 1993). It is coded as ? for *Macromyzon*.

Supplementary Figures

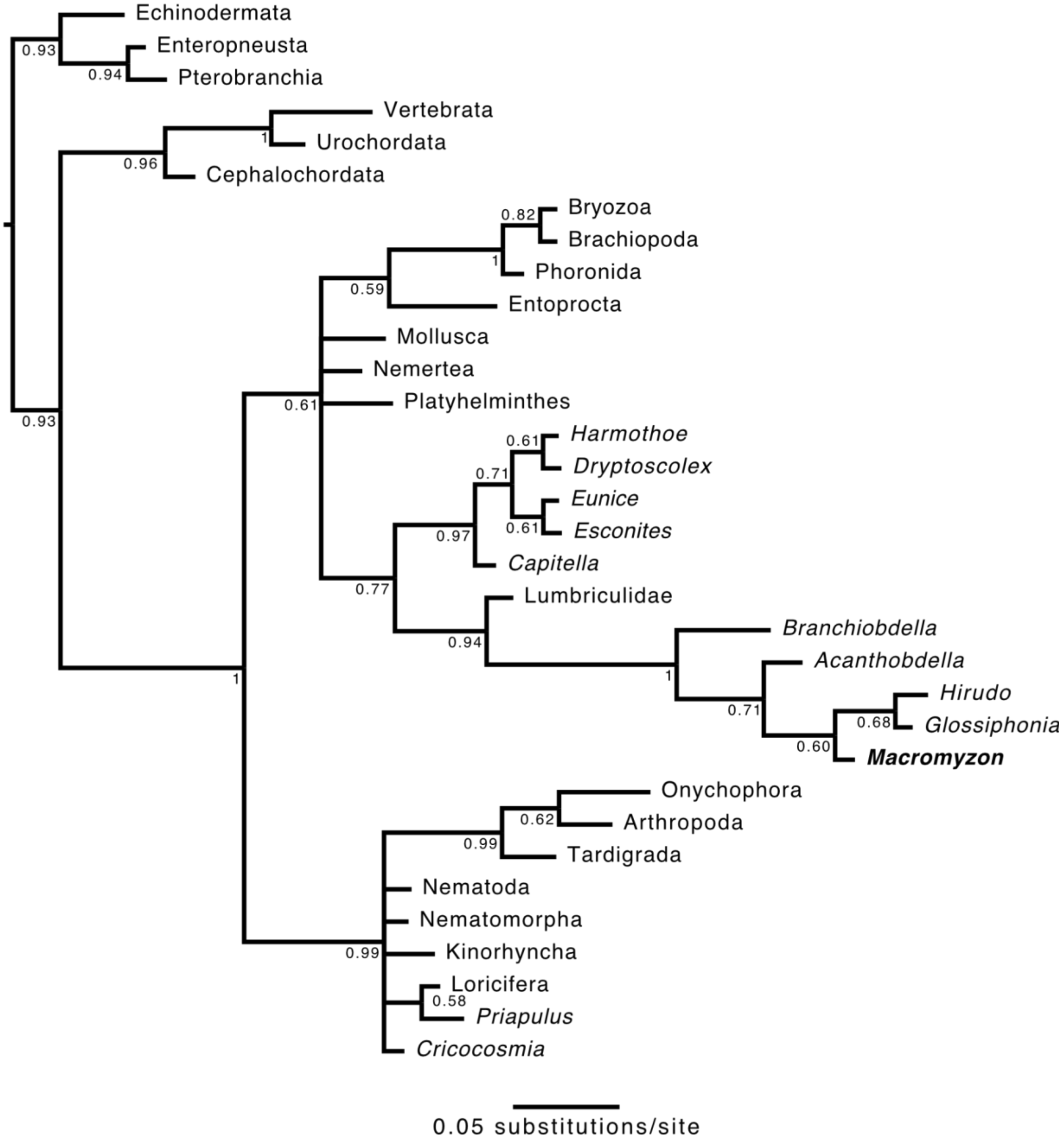

Figure S1. Bayesian inference phylogeny with posterior probability shown at each node. Raw output from this analysis is included as **Supplemental File 2**.

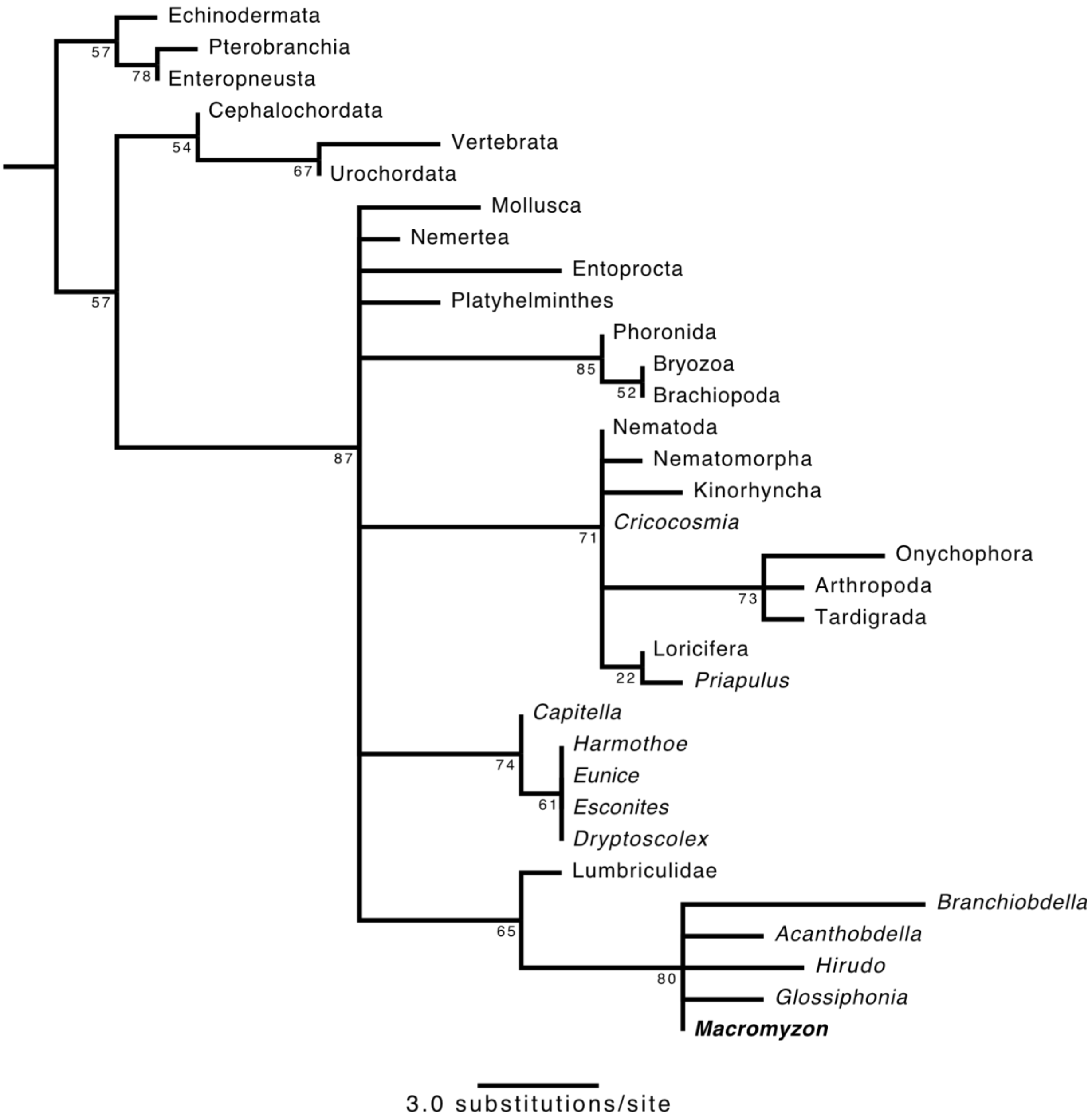

Figure S2. Maximum parsimony phylogeny with bootstrap support shown for all nodes. Raw output from this analysis is included as **Supplemental File 3**.

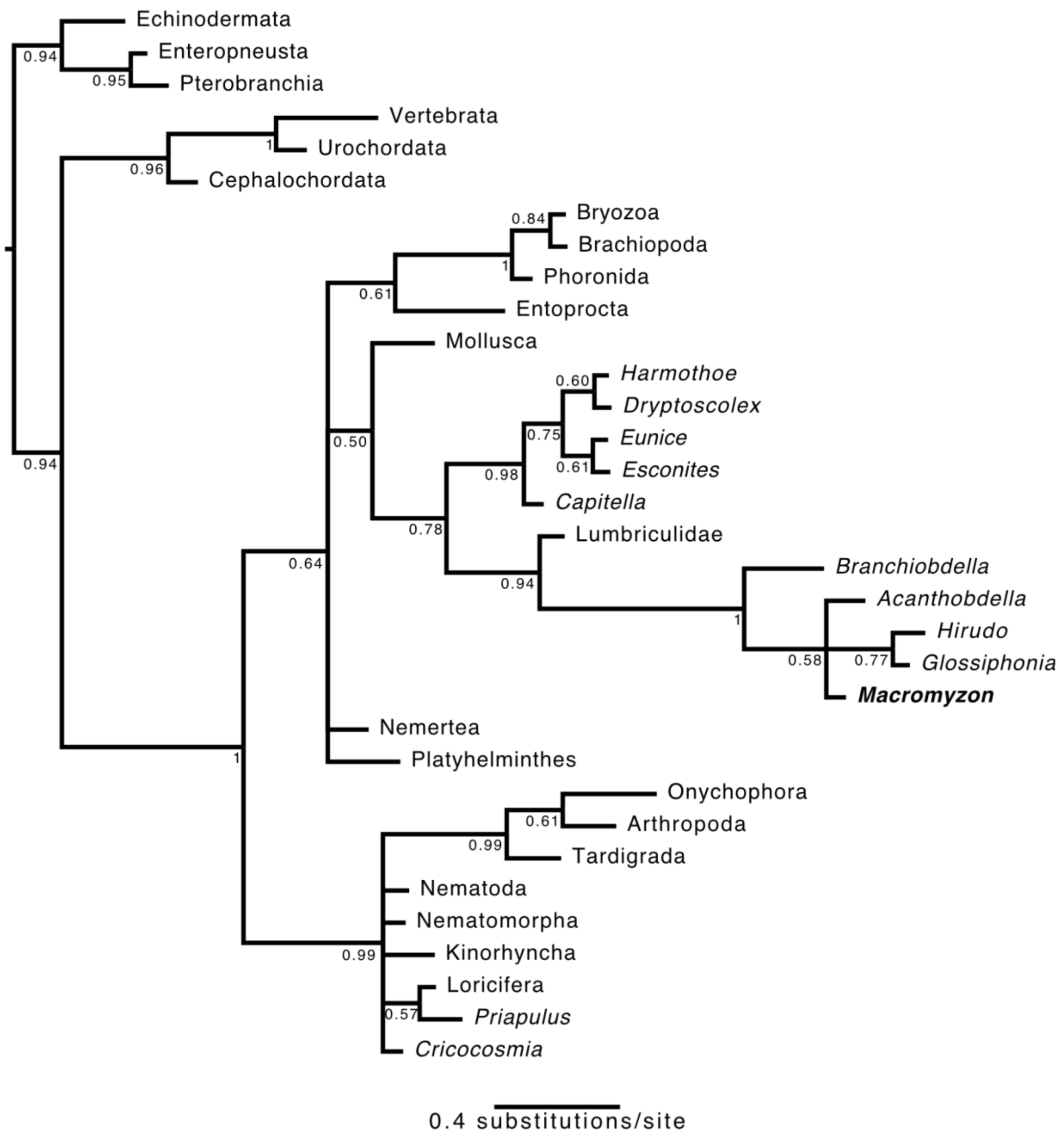

Figure S3. Bayesian inference phylogeny resulting from supplemental analysis in which characters 61 (segments externally subdivided into annuli) and 62 (annulation pattern in midbody segments) are both coded as "unknown" (?) for *Macromyzon siluricus*. In this analysis, *Macromyzon* is resolved as part of a polytomy containing *Acanthobdella* and Hirudinida (*i.e. Hirudo* and *Glossiphonia*); in the topology inferred from the original matrix, *Macromyzon* is sister to Hirudinida. Apart from that, there are some small discrepancies in relationships between spiralian taxa between analyses. Posterior probability is shown at all nodes. Raw output from this analysis is included as **Supplemental File 4**.

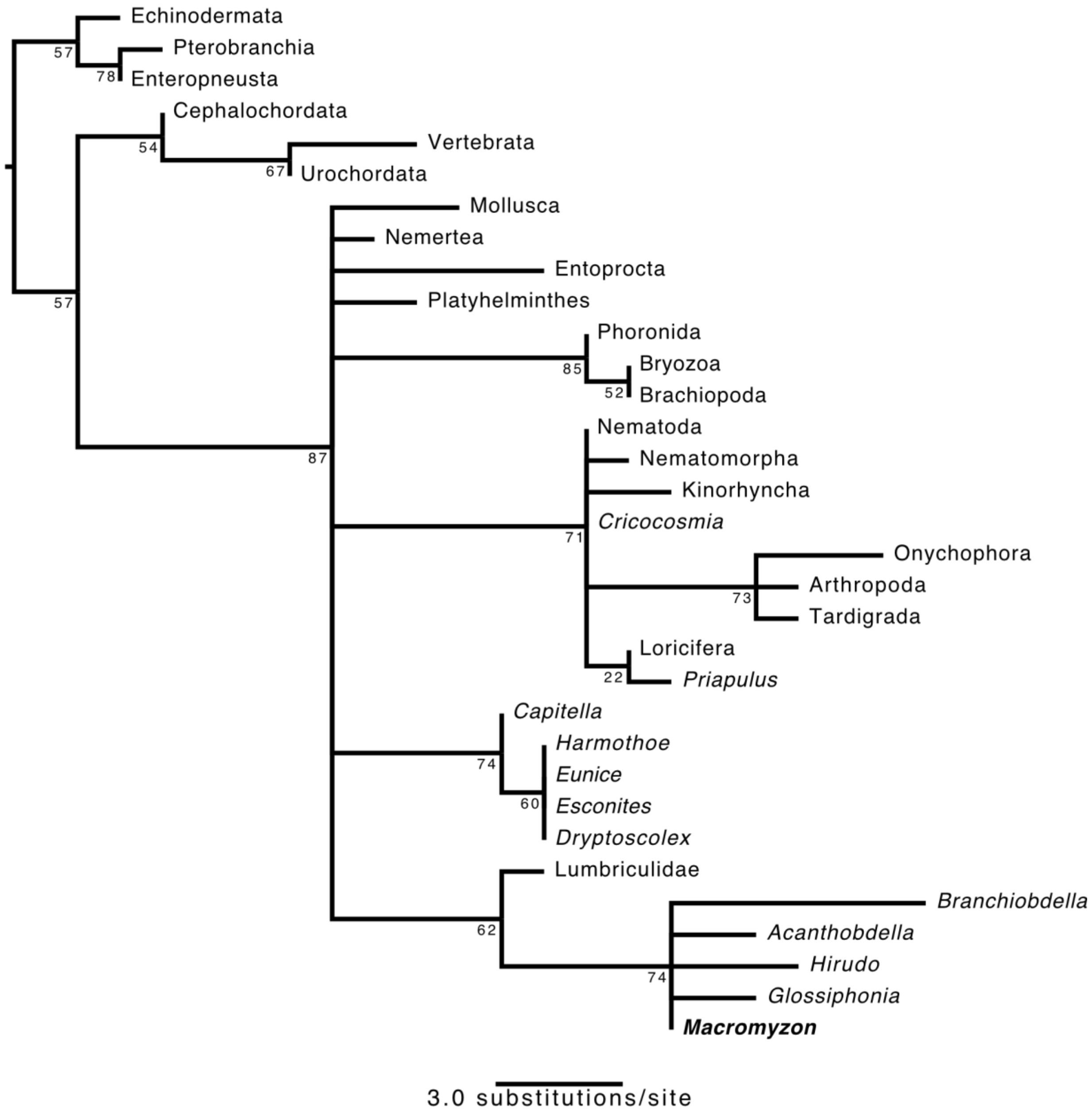

Figure S4. Maximum parsimony phylogeny resulting from supplemental analysis in which characters 61 (segments externally subdivided into annuli) and 62 (annulation pattern in midbody segments) are both coded as "unknown" (?) for *Macromyzon siluricus*. Topology does not differ from parsimony analysis on original matrix. Bootstrap support is shown at all nodes. Raw output from this analysis is included as **Supplemental File 5**.

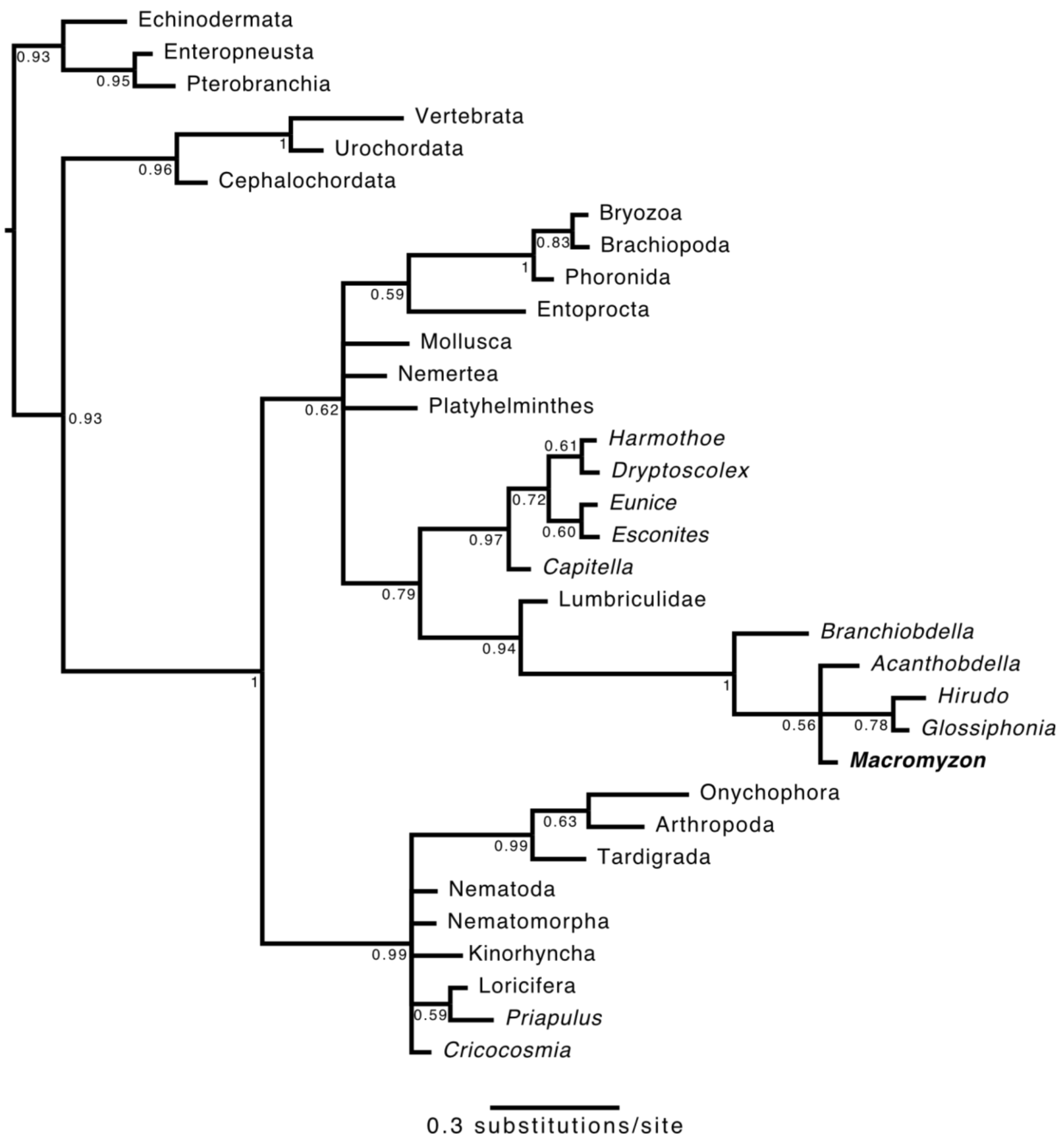

Figure S5. Bayesian inference phylogeny resulting from supplemental analysis in which character 62 (annulation pattern in midbody segments) is coded as "unknown" (?) for *Macromyzon siluricus*. In this analysis, *Macromyzon* is resolved as part of a polytomy containing *Acanthobdella* and Hirudinida (*i.e. Hirudo* and *Glossiphonia*); in the topology inferred from the original matrix, *Macromyzon* is sister to Hirudinida. The remainder of the resulting topology is the same in each analysis. Posterior probability is shown at all nodes. Raw output from this analysis is included as **Supplemental File 6**.

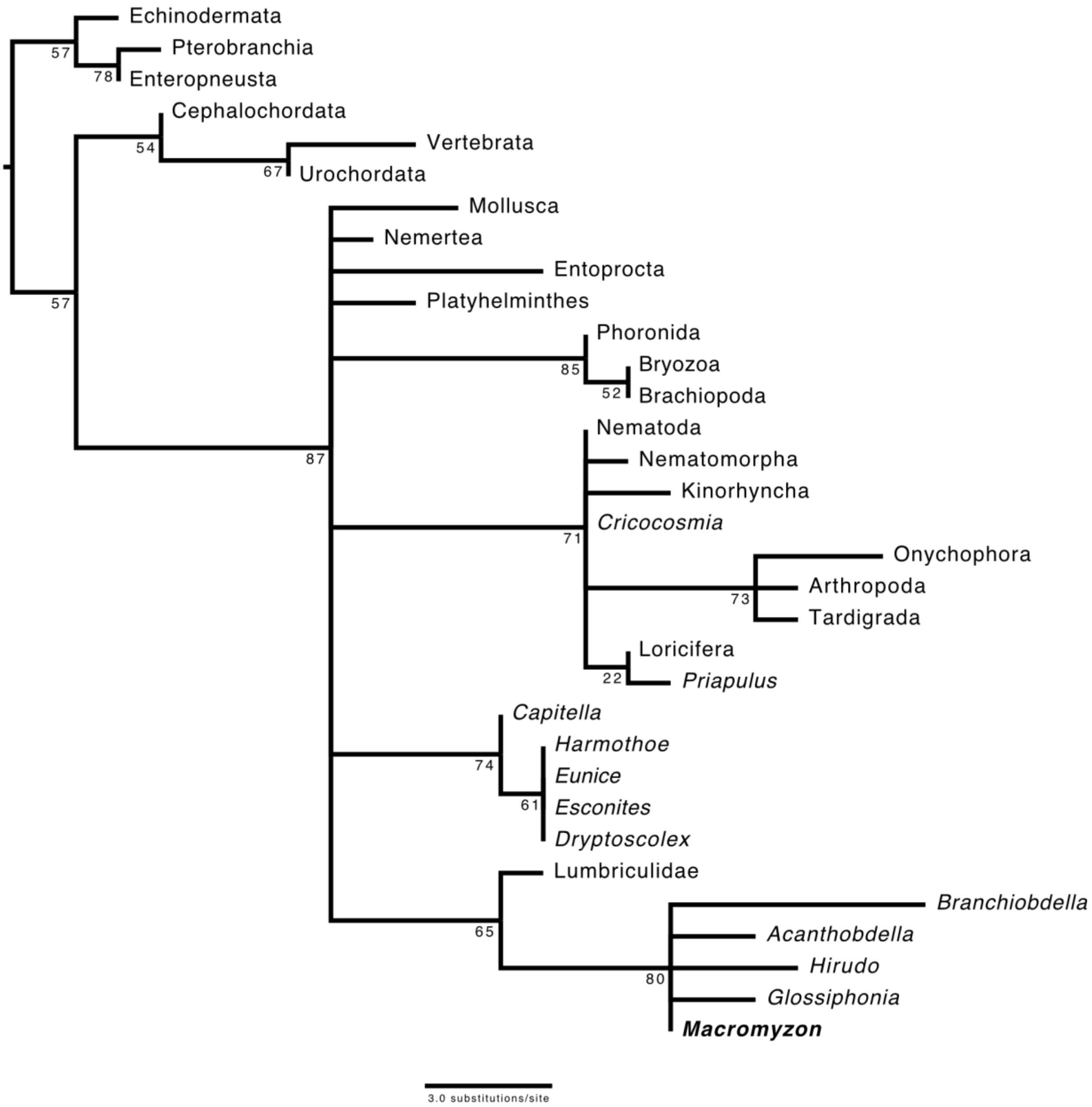

Figure S6. Maximum parsimony phylogeny resulting from supplemental analysis in which character 62 (annulation pattern in midbody segments) is coded as “unknown” (?) for *Macromyzon siluricus*. Topology does not differ from parsimony analysis on original matrix. Bootstrap support is shown at all nodes. Raw output from this analysis is included as **Supplemental File 7**.

References

Bielecki A, Cichocka JM, Jelen I, Swiatek P, Plachno BJ, Pikula D. 2014. New data about the functional morphology of the chaetiferous leech-like annelids Acanthobdella peledina (Grube, 1851) and Paracanthobdella livanowi (Ephstein, 1966) (Clitellata, Acanthobdellida). *Journal of Morphology*. DOI: 10.1002/jmor.20235.

de Carle DB, Gajda Ł, Bielecki A, Cios S, Cichocka JM, Golden HE, Gryska AD, Sokolov S, Shedko MB, Knudsen R, Utevsky S, Świątek P, Tessler M. 2022. Recent evolution of ancient Arctic leech relatives: systematics of Acanthobdellida. *Zoological Journal of the Linnean Society* 169:149–168. DOI: 10.1093/zoolinnean/zlac006.

Holt PC. 1965. The systematic position of the Branchiobdellidae (Annelida: Clitellata). *Systematic Zoology* 14:25–32.

Purschke G, Westheide W, Rohde D, Brinkhurst RO. 1993. Morphological reinvestigation and phylogenetic relationship of *Acanthobdella peledina* (Annelida, Clitellata). *Zoomorphology* 113:91–101. DOI: 10.1007/BF00403087.

Sawyer RT. 1986. *Leech Biology and Behaviour*. Oxford, UK: Clarendon Press.

Weigl AM. 1994. Ultrastructure of the adhesive organs in branchiobdellids (Annelida: Clitellata). *Transactions of the American Microscopical Society* 113:276–301. DOI: 10.2307/3226623.

Additional Supplemental Files

Supplemental File 1. (separate file)
Morphological matrix (in NEXUS format) used to infer evolutionary relationships of *Macromyzon siluricus*. Includes commands used to run phylogenetic analyses in Mr Bayes.

Supplemental File 2. (separate file)
Tree file containing Bayesian inference phylogeny. This tree is additionally shown in Fig.4A and S1.

Supplemental File3. (separate file)
Tree file containing maximum parsimony phylogeny. This tree is additionally shown in Fig. S2.

Supplemental File 4. (separate file)
Tree file containing Bayesian inference phylogeny resulting from supplemental analysis in which characters 61 (segments externally subdivided into annuli) and 62 (annulation pattern in midbody segments) are both coded as "unknown" (?) for *Macromyzon siluricus*. This tree is additionally shown in Fig. S3.

Supplemental File 5. (separate file)
Tree file containing maximum parsimony phylogeny resulting from supplemental analysis in which characters 61 (segments externally subdivided into annuli) and 62 (annulation pattern in midbody segments) are both coded as "unknown" (?) for *Macromyzon siluricus*. This tree is additionally shown in Fig. S4.

Supplemental File 6. (separate file)
Tree file containing Bayesian inference phylogeny resulting from supplemental analysis in which character 62 (annulation pattern in midbody segments) is coded as "unknown" (?) for *Macromyzon siluricus*. This tree is additionally shown in Fig. S5.

Supplemental File 7. (separate file)
Tree file containing maximum parsimony phylogeny resulting from supplemental analysis in which character 62 (annulation pattern in midbody segments) is coded as "unknown" (?) for *Macromyzon siluricus*. This tree is additionally shown in Fig. S6.
